# m^6^A-Modified GATA2 Enhances Odontogenic Differentiation in Stem Cells from the Apical Papilla

**DOI:** 10.3390/ijms26072920

**Published:** 2025-03-24

**Authors:** Haoqing Yang, Fengning Yuan, Jiaxin Song, Yishu Huang, Zhaochen Shan, Zhipeng Fan

**Affiliations:** 1 Outpatient Department of Oral and Maxillofacial Surgery, School of Stomatology, Capital Medical University, Beijing 100070, China; yanghaoqing@mail.ccmu.edu.cn (H.Y.);; 2 Laboratory of Molecular Signaling and Stem Cells Therapy, Beijing Key Laboratory of Tooth Regeneration and Function Reconstruction, School of Stomatology, Capital Medical University, Beijing 100070, China; 3 Beijing Laboratory of Oral Health, Capital Medical University, Beijing 100054, China

**Keywords:** GATA2, m^6^A modification, odontogenic differentiation, SCAPs

## Abstract

Epigenetic modifications play a crucial role in regulating stem cell differentiation. Among these, N6-methyladenosine (m^6^A) modification significantly impacts mRNA stability and translation. However, its role in dental stem cell differentiation remains largely unexplored. Functional assays, including ALP activity, alizarin red S staining, qPCR, and Western blot, were conducted to assess odontogenic differentiation. Then, an in vivo dentin formation model was used to validate our findings. Additionally, we employed RNA stability assays and m^6^A site mutagenesis to investigate the regulatory mechanism of m^6^A modification in GATA2-mediated differentiation. Our results demonstrated that overexpression of GATA2 significantly promoted SCAP odontogenic differentiation. Moreover, in vivo studies confirmed that GATA2 overexpression enhances dentin formation in mouse models. Conversely, knockdown of GATA2 or mutation of its m^6^A sites led to reduced mRNA stability and decreased odontogenic differentiation. m^6^A modification is enriched in the 3′ untranslated region (3′UTR) of GATA2 mRNA, regulating its stability and expression. Our findings indicate that m^6^A modification contributes to the post-transcriptional regulation of GATA2, enhancing its stability and promoting SCAP-mediated odontogenic differentiation and dentin formation. This study provides new insights into the epigenetic regulation of dental stem cells and suggests a potential molecular target for dental tissue regeneration.

## 1. Introduction

Dental injuries stimulate inflammatory and regenerative cellular responses in pulp tissue [1]. However, the physiological dentin regeneration proceeds at a slow rate, initiating approximately three months post-lesion. Thus, identifying the key genes that regulate the formation of dentin by dental stem cells is crucial in order to accelerate the generation of dentin. Stem cells from the apical papilla are the precursor cells to odontoblasts, which directly form dentin. Compared with other dental stem cells including dental pulp stem cells (DPSCs), SCAPs have a higher mineralization potential and proliferation rate. Also, the apical papilla and SCAPs can be easily isolated after tooth extraction [2,3]. They may restore dental defects more effectively and are characterized by ease of expansion in vitro, as well as minimal immunogenicity [4,5]. Therefore, we chose to conduct experiments with SCAPs to identify molecular factors that regulate the odontogenic differentiation of SCAPs.

Transcription factor regulation, a key mechanism in gene expression control, involves the binding of transcription factors to specific DNA sequences (e.g., promoters, enhancers) to directly modulate target gene transcription. GATA factors are transcriptional regulators present in animals, which recognize the DNA sequence W-G-A-T-A-R through a single type IV zinc finger. Six GATA transcription factors can be identified in humans (GATA1 through GATA6). They are required for essential developmental processes such as the differentiation of the hematopoietic and central nervous systems or of embryonic stem cells and cardiovascular embryogenesis [6,7]. Dysregulation of GATA proteins has been linked to bone marrow failure and blood diseases [8,9,10,11]. GATA2, a pioneer transcription factor, can bind to heterochromatic DNA regions that are inaccessible to other factors, facilitating chromatin decondensation and enabling subsequent transcriptional regulation. Additionally, GATA2 cooperates with other transcription factors such as GATA1, CEBPA, and SPI1 (PU.1) to target specific genes or microRNAs [12,13,14]. In the bone marrow, GATA2 is expressed in HSCs, HPCs, erythroid precursors, and eosinophilic and megakaryocytic progenitors, and is essential for myeloid lineage commitment in HSCs/HPCs [15]. Though previous studies have indicated that GATA2 is associated with the development and differentiation of bone marrow stem cells, the focus has primarily been on hematopoietic stem cells, with no research on dental stem cells [16]. Therefore, we aim to investigate the role and regulatory mechanisms of GATA2 in SCAPs.

Besides GATA2’s functional significance, understanding how its expression is modulated is crucial. During the process of dentin regeneration, epigenetic regulation plays a significant role, such as histone modification, DNA modification, and mRNA modification. m^6^A RNA methylation is one of the most prevalent post-transcriptional modifications, accounting for nearly 50% of all methylated ribonucleotides [17,18]. It is a crucial regulator of mRNA export, splicing, translation, and turnover [19,20]. Previous studies indicate that reducing m^6^A levels impairs embryonic stem cell differentiation, showing its key role in stem cell growth and differentiation [21]. Methyltransferase-like 3 (METTL3) is the originally characterized methyltransferase that catalyzes m^6^A modifications [22]. Previous studies indicated that METTL3 modulated dynamic m^6^A levels, which regulated stem cell fate, mammalian development, and carcinogenesis [23,24,25,26]. Han et al. reported that METTL3 m^6^A methylation inhibits DGCR8-mediated miR-196b-5p maturation, promoting SCAP odontogenic differentiation [27]. In addition, Tian et al. found inhibiting the glutamine–αKG axis affects IGF2 m^6^A methylation, regulating odontogenic differentiation of mesenchymal stem cells [28]. Following previous studies, we conducted m^6^A-seq on SCAP cells and observed significant enrichment of m^6^A modifications in the 3′UTR of GATA2. This prompted us to investigate whether m^6^A modification acts as a key regulatory mechanism for GATA2 in odontogenic differentiation.

In this study, we explored the role and underlying mechanism of GATA2 in influencing the odontogenesis differentiation capability by using SCAPs. We discovered that GATA2 promoted odontogenic differentiation of SCAPs and SCAPs-mediated dentin regeneration. Mechanistically, we found that the 3′UTR of GATA2 contains m^6^A modification sites, and mutations in this region result in a weakened ability to promote odontogenesis in SCAPs.

## 2. Results

### 2.1. GATA2 Enhances the Odontogenic Differentiation of SCAPs

**Figure 1:**
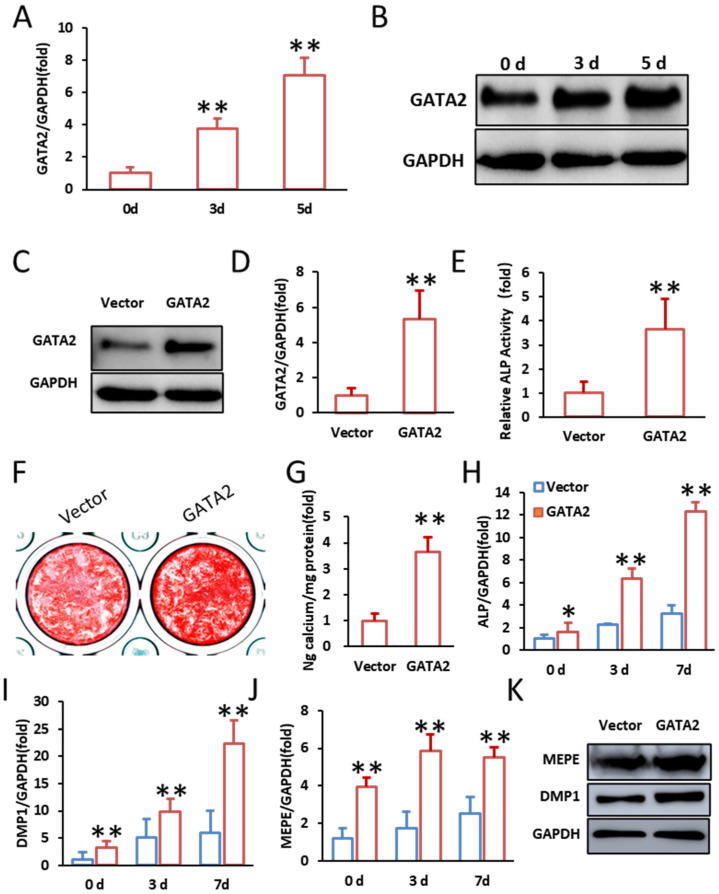
Overexpression of GATA2 promotes odontogenic differentiation of SCAPs in vitro. (**A**) qPCR revealed increased GATA2 expression in SCAPs on days 0, 3, and 5. (**B**) Western blot analysis showed elevated GATA2 protein in SCAPs at days 0, 3, and 5. (**C**) Western blot confirms GATA2 overexpression in SCAPs. (**D**) qPCR shows significant increase in GATA2 mRNA in GATA2 overexpression group. (**E**) ALP activity assay showing a marked increase in SCAPs overexpressing GATA2. (**F**,**G**) Alizarin red S staining and quantification of calcium deposition illustrating increased mineralized nodule formation in the GATA2-overexpression group. (**H**–**J**) qPCR analysis of ALP, DMP1, and MEPE expression at 0, 3, and 7 days post-odontogenic induction showed significantly increase in SCAPs with GATA2 overexpression. (**K**) Western blot shows upregulation of MEPE and DMP1 in SCAPs overexpressing GATA2. GAPDH served as the internal control. Student’s *t*-test and one-way ANOVA were performed to determine statistical significance. All error bars represent the SD (*n* = 3 or 6). * *p* ≤ 0.05, ** *p* ≤ 0.01.

Firstly, we explored the expression pattern of GATA2 during the odontogenic differentiation of SCAPs. Quantitative PCR (qPCR) analysis showed a progressive increase in GATA2 expression from day 0 to day 5 during odontogenic differentiation (Figure 1A). Western blot analysis corroborated this trend, demonstrating elevated GATA2 protein levels at days 3 and 5 post-induction (Figure 1B). These results indicate that GATA2 expression is dynamically regulated during SCAP differentiation.

To further explore the role of GATA2 in the odontogenic differentiation of SCAPs, we employed lentiviral vectors to overexpress and knockdown GATA2. Successful transduction was confirmed by Western blot and qPCR analysis, demonstrating significant overexpression of GATA2 in the overexpression group (Figure 1C,D). Overexpression of GATA2 promoted the odontogenic differentiation of SCAPs. This was evidenced by a significant increase in alkaline phosphatase (ALP) activity on day 3 of mineralization induction (Figure 1E). Enhanced mineral deposition was confirmed by more extensive alizarin red staining and quantification on day 14 (Figure 1F,G). Furthermore, the expression levels of key odontogenic markers, including ALP, DMP1, and MEPE, were upregulated at 0, 3, and 7 days post-induction, as detected by qPCR (Figure 1H–J). Western blot analysis further confirmed elevated protein levels of MEPE and DMP1 in the GATA2-overexpression group at 14 days (Figure 1K).

**Figure 2:**
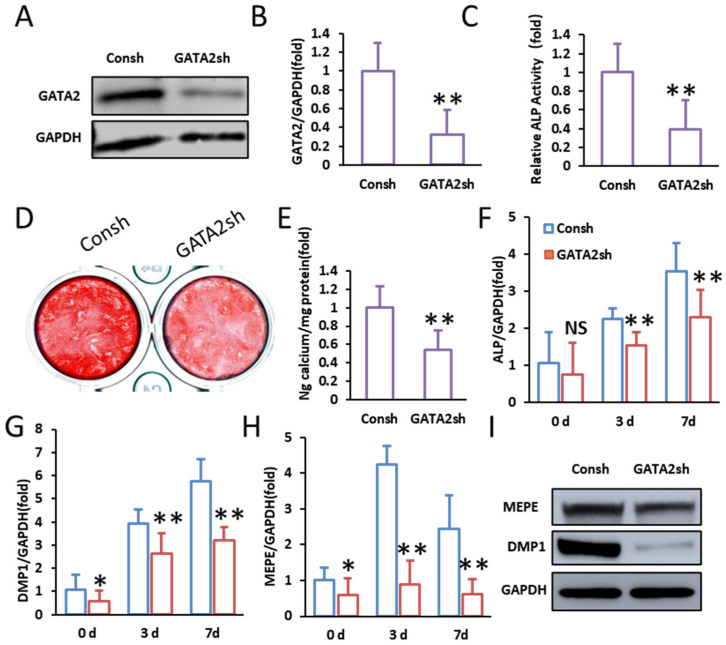
Knockdown of GATA2 inhibits odontogenic differentiation of SCAPs in vitro. (**A**) Western blot showed reduced GATA2 protein in SCAPs upon silencing. (**B**) qPCR showed significantly decreased GATA2 mRNA in SCAPs upon silencing. (**C**) ALP activity was significantly reduced in SCAPs upon GATA2 silencing. (**D**,**E**) Alizarin red staining and quantification of calcium deposition showed decreased mineralization in SCAPs upon GATA2 silencing. (**F**–**H**) qPCR showed significant downregulation of ALP, DMP1, and MEPE in SCAPs upon GATA2 silencing at days 0, 3, and 7. (**I**) Western blot showed decreased MEPE and DMP1 protein in SCAPs upon GATA2 silencing. GAPDH served as the internal control. Student’s *t*-test was performed to determine statistical significance. All error bars represent the SD (*n*= 3). NS *p* > 0.05, * *p* ≤ 0.05, ** *p* ≤ 0.01.

Conversely, the knockdown of GATA2 impaired the odontogenic differentiation of SCAPs. Successful knockdown of GATA2 was confirmed by Western blot and qPCR, demonstrating markedly reduced GATA2 expression in the knockdown group (Figure 2A,B). Cells with GATA2 silencing exhibited significantly reduced ALP activity on day 3 of mineralization induction (Figure 2C). Mineralization ability was further suppressed, as indicated by decreased alizarin red staining and quantification on day 14 (Figure 2D,E). Consistently, the expression of odontogenic markers ALP, DMP1, and MEPE was significantly downregulated at 0, 3, and 7 days post-induction in the GATA2-knockdown group (Figure 2F–H). Western blot analysis confirmed lower protein levels of MEPE and DMP1 in the knockdown group on day 14 (Figure 2I). These findings indicate that GATA2 acts as a positive regulator of SCAP odontogenic differentiation.

### 2.2. GATA2 Promotes Dentin Formation In Vivo

**Figure 3:**
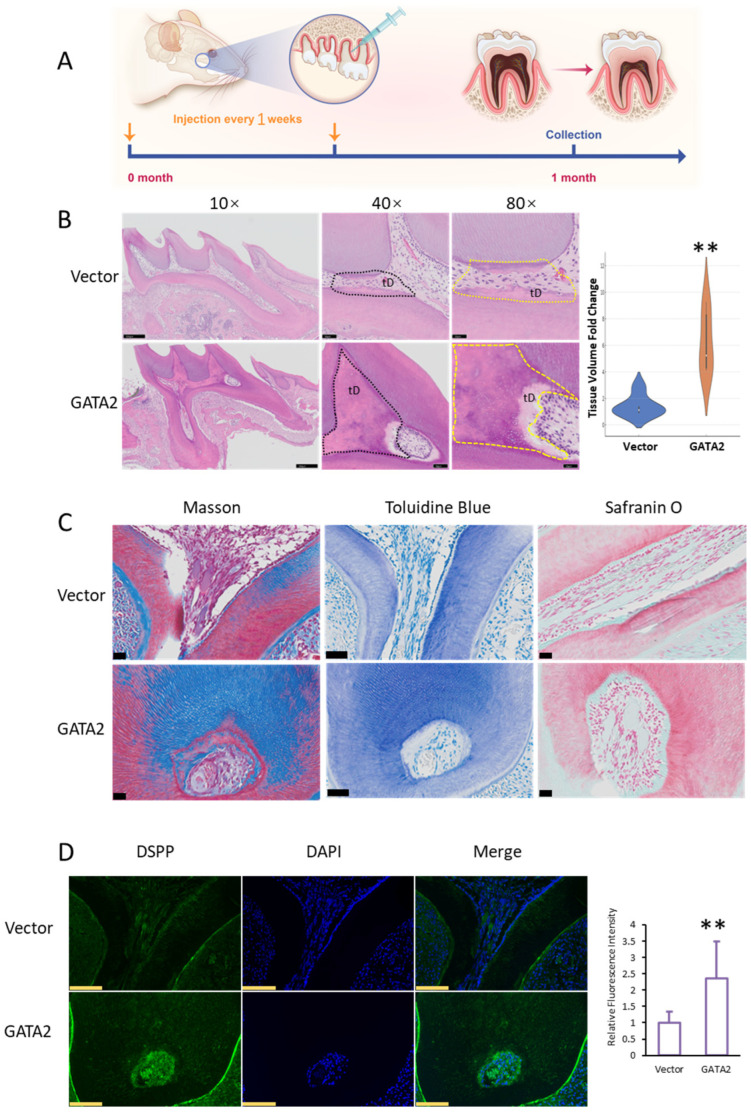
GATA2 promotes dentin formation in vivo. (**A**) Schematic illustration of adenoviral vector injection into the buccal apical region of the mouse first molar for gene delivery. (**B**) Representative HE-stained sections of the first molar region in the GATA2 and control groups. Sequential magnifications (10×, 40×, 80×) show a thicker predentin layer and better odontoblast alignment in the GATA2 group. High-magnification images label newly formed dentin as “Tertiary dentin (tD)”. Black dashed lines outline tertiary dentin boundaries, and yellow dashed lines highlight regions of tertiary dentin formation for quantitative analysis. Scale bars: 250 µm, 50 µm, and 25 µm. The violin plot quantifies dentin volume, revealing a significant increase in the GATA2 group. (**C**) Masson’s trichrome (**left**), toluidine blue (**middle**), and safranin O (**right**) staining reveal enhanced collagen deposition, mineralized tissue formation, and proteoglycan distribution in the GATA2 group compared to the control group. Scale bars: 50 µm. (**D**) Immunofluorescence staining for DSPP demonstrates stronger odontoblast activity in the GATA2 group, as shown by increased DSPP expression. Scale bars: 100 µm. Quantification of fluorescence intensity is shown on the right. Statistical significance was determined using Student’s *t*-test. Error bars represent standard deviation (SD) from three independent experiments. ** *p* ≤ 0.01.

To assess the role of GATA2 in dentin regeneration, we utilized adenoviral vectors to introduce genes into the buccal region of the mouse first molar (Figure 3A). Following gene delivery, the impact of GATA2 overexpression on dentin formation within the dental pulp was evaluated.

H&E staining of the first molar region revealed significant differences between the GATA2-overexpression and control groups. In the GATA2 group, thicker predentin layers and better-organized odontoblast alignment were observed compared to the control group (Figure 3B). Quantification of regenerated tissue volume using a violin plot showed a significant increase in the GATA2 group compared to the control group, indicating enhanced dentin formation (Figure 3B, Supplementary Figure S1). Masson’s trichrome, toluidine blue, and safranin O staining provided further evidence of dentin regeneration. In the GATA2 group, collagen deposition, mineralized tissue formation, and proteoglycan distribution were notably enhanced compared to the control group, suggesting improved extracellular matrix formation and tissue maturation (Figure 3C).

To confirm the functional activity of odontoblasts, immunofluorescence staining for DSPP, a key marker of dentinogenesis, was performed. The GATA2-overexpression group exhibited significantly stronger DSPP expression compared to the control group, supporting the conclusion that GATA2 enhances odontoblast activity and promotes dentin formation. Quantitative analysis of DSPP fluorescence intensity further validated this finding (Figure 3D).

These results collectively demonstrate that GATA2 promotes dentin formation in vivo by enhancing odontoblast activity, collagen deposition, and extracellular matrix formation.

### 2.3. Identification of m^6^A Modification in GATA2

**Figure 4:**
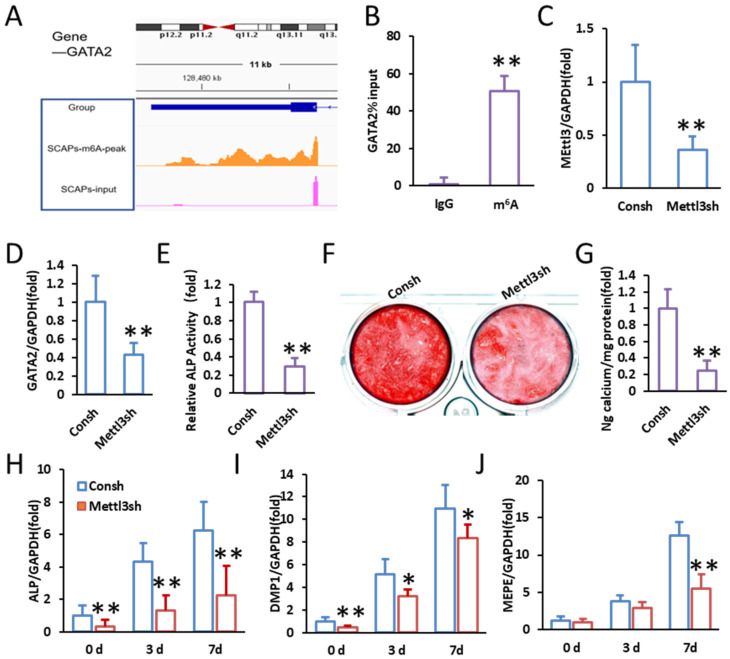
GATA2 expression increases with SCAP development by m^6^A modification. (**A**) MeRIP-seq data showing m^6^A modification peaks in the 3′ untranslated region (UTR) of GATA2 in SCAPs. (**B**) MeRIP-qPCR confirming the enrichment of m^6^A on GATA2 mRNA in SCAPs. (**C**) qPCR analysis showing effective silencing of METTL3 in SCAPs using shRNA. (**D**) qPCR results showing a significant reduction in GATA2 expression upon METTL3 silencing in SCAPs. (**E**) ALP activity assay showing a marked decrease in METTL3-silenced SCAPs. (**F**,**G**) Alizarin red S staining and quantification of calcium deposition demonstrated decreased mineralized nodule formation in METTL3-silenced SCAPs. (**H**–**J**) qPCR analysis of ALP, DMP1, and MEPE expression at 0, 3, and 7 days post-odontogenic induction showed a significant decrease in METTL3-silenced SCAPs. GAPDH served as the internal control. Student’s *t*-test and one-way ANOVA were performed to determine statistical significance. All error bars represent the SD (*n* = 3). * *p* ≤ 0.05, ** *p* ≤ 0.01.

To determine whether GATA2 undergoes m^6^A modification, we analyzed m^6^A-seq (MeRIP-seq) data, which revealed significant m^6^A enrichment in the 3′ untranslated region (3′UTR) of GATA2 in SCAPs. Visualization using the Integrative Genomics Viewer (IGV) further confirmed the presence of prominent m^6^A peaks, indicating that GATA2 transcripts are subject to m^6^A modification (Figure 4A). To further validate this observation, MeRIP-qPCR was performed, which confirmed the enrichment of m^6^A-modified GATA2 mRNA in SCAPs (Figure 4B).

To investigate the regulatory mechanism underlying m^6^A modification of GATA2, we further investigated the role of METTL3, an m^6^A writer protein, in regulating GATA2 expression by performing METTL3 knockdown in SCAPs. qPCR analysis confirmed a significant reduction in METTL3 mRNA levels upon transfection with METTL3 shRNA (Mettl3sh), indicating efficient knockdown (Figure 4C). Notably, the suppression of METTL3 resulted in a marked decrease in GATA2 expression, suggesting that METTL3 positively regulates GATA2 at the post-transcriptional level (Figure 4D).

To assess the functional impact of METTL3 depletion on odontogenic differentiation, we performed an ALP activity assay, which showed a marked decrease in ALP activity in METTL3-knockdown SCAPs (Figure 4E). Additionally, alizarin red S staining demonstrated reduced calcium deposition in METTL3-silenced cells, indicating impaired mineralization (Figure 4F,G). Further analysis of odontogenic marker gene expression revealed that METTL3 knockdown significantly downregulated ALP, DMP1, and MEPE expression at 0, 3, and 7 days post-induction (Figure 4H–J).

These findings indicate that m^6^A modification by METTL3 is essential for maintaining GATA2 expression and promoting SCAP odontogenic differentiation.

### 2.4. Mutation of m^6^A Modification Sites Reduces GATA2 Stability and Impairs Odontogenic Differentiation

**Figure 5:**
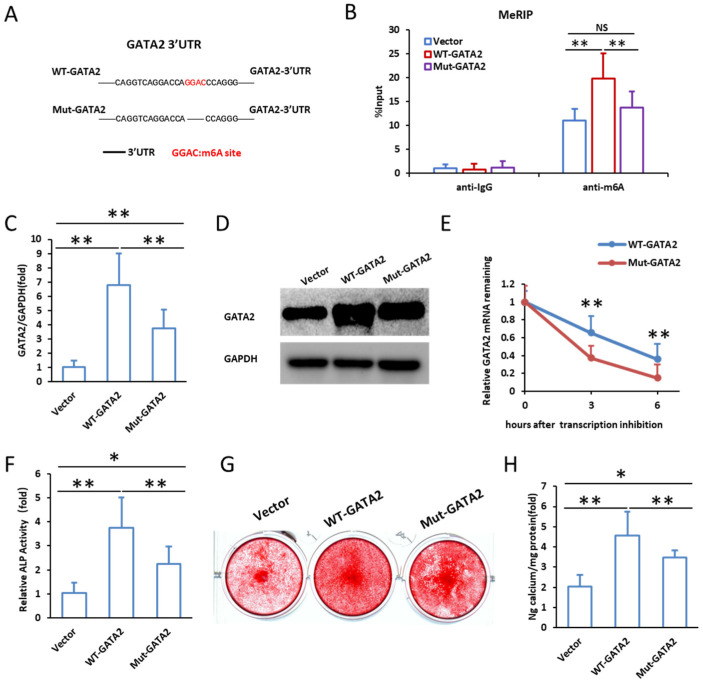
Mutation of the m^6^A modification site in GATA2 3′UTR reduces GATA2 stability and impairs odontogenic differentiation. (**A**) The schematic diagram illustrates the wild-type and mutant versions of the GATA2 3′UTR. (**B**) MeRIP-qPCR shows that WT-GATA2 has significantly higher m^6^A enrichment than vector, while Mut-GATA2 has lower m^6^A levels. (**C**) qPCR shows that GATA2 mRNA expression is significantly lower in Mut-GATA2 than in WT-GATA2. (**D**) Western blot confirms that GATA2 protein expression is significantly reduced in Mut-GATA2 compared to WT-GATA2. (**E**) RNA stability assays show significantly faster GATA2 mRNA degradation in Mut-GATA2 than in WT-GATA2. (**F**) ALP activity is significantly higher in WT-GATA2-transfected SCAPs than in vector controls. (**G**) Representative images of alizarin red S staining show enhanced mineral deposition in the WT-GATA2 group compared to the vector and Mut-GATA2 groups. (**H**) Quantification of calcium deposition reveals significantly reduced mineralization in the Mut-GATA2 group compared to WT-GATA2. GAPDH serves as the internal control. Statistical significance was determined using Student’s *t*-test. Error bars represent standard deviation (SD) from three independent experiments. NS *p* > 0.05, * *p* ≤ 0.05, ** *p* ≤ 0.01.

Sequence analysis identified a conserved m^6^A modification site (GGAC) within the 3′UTR of GATA2, which may play a crucial role in its post-transcriptional regulation (Supplementary Data S1). To investigate the functional impact of m^6^A modification on GATA2, we generated lentiviral constructs expressing either wild-type GATA2 (WT-GATA2) or a mutant form of GATA2 with disrupted m^6^A sites (Mut-GATA2). The schematic representation of these constructs is shown in Figure 5A. To confirm successful transfection and expression, we performed MeRIP-qPCR, qPCR, and Western blot analysis. MeRIP-qPCR demonstrated a significant enrichment of m^6^A modification in WT-GATA2, whereas Mut-GATA2 showed markedly reduced m^6^A levels, confirming the disruption of m^6^A modification (Figure 5B). qPCR analysis revealed that the mRNA expression of GATA2 was significantly lower in the Mut-GATA2 group compared to the WT-GATA2 group (Figure 5C), and Western blot results further confirmed that GATA2 protein levels were markedly reduced in Mut-GATA2-expressing cells (Figure 5D). To examine whether m^6^A modification affects GATA2 mRNA stability, we conducted Actinomycin D chase assays. The results showed that the degradation rate of GATA2 mRNA was significantly accelerated in the Mut-GATA2 group compared to the WT-GATA2 group, indicating that m^6^A modification plays a critical role in stabilizing GATA2 transcripts (Figure 5E). Next, we assessed the effect of m^6^A modification on the odontogenic differentiation potential of SCAPs. ALP activity was measured on day 3 after mineralization induction, revealing that the WT-GATA2 group exhibited significantly higher ALP activity compared to the vector control, while the Mut-GATA2 group showed a notable reduction in ALP activity (Figure 5F). To further evaluate mineralization, alizarin red S staining was performed on day 14, which showed enhanced mineral deposition in the WT-GATA2 group, whereas the Mut-GATA2 group exhibited significantly impaired mineralization (Figure 5G). Quantification of calcium deposition confirmed a significant decrease in mineralization in the Mut-GATA2 group compared to WT-GATA2 (Figure 5H). These findings suggest that m^6^A modification of GATA2 is essential for its stability and functional role in promoting the odontogenic differentiation of SCAPs. Mutation of the m^6^A sites disrupts this modification, leading to reduced GATA2 stability and impaired differentiation capacity.

## 3. Discussion

GATA2 is critical for stem cell differentiation [15]. However, its specific effects on dentin regeneration, along with the underlying regulatory mechanisms, remain to be fully elucidated. Our in vitro studies show that GATA2 enhances odontogenic differentiation of SCAPs, as indicated by increased ALP activity, ARS staining, and upregulation of key odontogenic markers (Figure 2). Consistent with these findings, in vivo experiments confirm that GATA2 promotes dentin formation in SCAPs, supported by H&E staining, Masson staining, toluidine blue staining, Safranin O staining, and DSPP immunofluorescence (Figure 5). These results prompt further investigation into the regulatory mechanisms of GATA2.

In addition to in vitro experiments, validation through in vivo studies is also crucial. In dentin regeneration and formation studies, common models include nude mouse subcutaneous implantation models, rat dental pulp defect models, and minipig pulp regeneration models, each offering unique advantages [29,30]. However, this study selected the first molar model of mice for GATA2 functional validation based on the following considerations: Firstly, mice possess the advantages of a well-defined genetic background and experimental accessibility, enabling precise regulation of GATA2 expression through gene editing technology. Secondly, the smaller pulp chamber in mice aligns with the requirements for refined genetic manipulations and allows observation of dental pulp tissue formation within a shorter timeframe. Additionally, the relatively short repair cycle of mouse dental pulp injury (approximately 4 weeks) facilitates the evaluation of GATA2’s effects on dentin formation within a limited experimental period while achieving a larger sample size (n = 8) to enhance statistical power. In contrast, although rat and minipig models better resemble human dental pulp anatomy, they involve prolonged experimental cycles and higher costs. Future studies should further validate GATA2’s function in large animal models to assess its therapeutic potential in clinical pulp regeneration.

This study achieved GATA2 overexpression via Adeno-associated virus 9 (AAV9)-mediated delivery targeting the dental pulp tissue within the pulp chamber. While SCAPs are localized to the apical papilla of developing teeth, the adult dental pulp harbors a heterogeneous mix of cells (e.g., DPSCs, odontoblasts, mesenchymal cells), and biological distinctions may exist between SCAPs and adult pulp cell populations. However, existing evidence indicates that SCAPs share highly similar odontogenic differentiation capabilities with dental pulp-derived mesenchymal stem cells, and SCAPs can exhibit DPSC-like dentinogenic potential under appropriate microenvironmental conditions [31]. Furthermore, AAV9 demonstrates robust tissue penetrability, enabling efficient infection of diverse cell types within the pulp chamber, such as DPSCs and odontoblasts, thereby effectively modulating GATA2 expression and promoting dentin regeneration. Therefore, this study employed SCAPs for in vitro investigation combined with AAV9’s high-efficiency gene delivery capacity in dental pulp tissue to comprehensively evaluate the role of GATA2 in dentin regeneration.

Adeno-associated virus has been widely used in the field of gene therapy due to its low immunogenicity, long-term gene expression, and broad tissue tropism. Among various serotypes, AAV9 has been reported to have strong tropism for bone and hard tissues, making it suitable for studies promoting osteogenesis and dentinogenesis [32]. Previous studies have demonstrated that AAV9 can efficiently infect bone marrow mesenchymal stem cells (BMSCs) and dental stem cells, achieving high-level gene expression in dental pulp tissue [33]. Compared with other serotypes (e.g., AAV2 and AAV5), AAV9 exhibits higher transduction efficiency and tissue penetration in hard tissue environments, rendering it an ideal vector for dentin regeneration research. In this study, the AAV9 vector was employed to mediate the expression of GATA2, primarily based on its stability in the dental pulp microenvironment and its efficient infection of SCAPs (stem cells from the apical papilla). Additionally, AAV9 has been extensively applied in dentin repair, with its superior transduction ability facilitating enhanced gene delivery efficiency. This enables a more precise assessment of the regulatory effects of GATA2 on SCAP differentiation and dentin regeneration. Future research could further optimize the AAV9 delivery system to improve its specificity and safety, thereby promoting the clinical translation of GATA2 in the field of dentin regeneration.

Following the functional validation of GATA2, we probed its regulatory mechanisms. In the post-transcriptional regulation of eukaryotic mRNA, the 3′UTR plays a critical role in determining mRNA stability, degradation rate, and translation efficiency, all of which can be influenced by RNA modifications [34,35,36,37]. Among these modifications, the m^6^A modification stands out as the most abundant and dynamic, exerting regulatory control over gene expression. In particular, the m⁶A modification of mRNA exerts a significant influence on the differentiation and aging of stem cells [38]. Xu et al. reported the m^6^A demethylase FTO promotes odontogenic differentiation of SCAPs by stabilizing RBM4 mRNA [39]. In addition, Pan et.al reported METTL3 enhances the odontogenic differentiation of dental pulp stem cells via increasing GDF6 and STC1 mRNA stability [40]. Our study demonstrates that METTL3 stabilizes GATA2 mRNA via m^6^A deposition in its 3′UTR. Knockdown of METTL3 or mutation of 3′UTR m^6^A motifs reduced GATA2 expression by accelerating mRNA decay, thereby impairing odontogenic differentiation of SCAPs and dentin formation. This METTL3-m^6^A-GATA2 axis constitutes a critical regulatory mechanism for dental stem cell differentiation.

Our findings highlight GATA2 as a potential target for dental tissue regeneration. Given that m^6^A modification is widespread and our previous research has shown its role in WJCMSCs’ osteogenic differentiation [41], we therefore hypothesize that GATA2—m^6^A plays a role in a broader cell differentiation range. Future studies should explore GATA2-based gene therapy in other cells and large animal models to assess its feasibility in clinical applications. Advanced gene delivery methods, such as adenoviral or CRISPR-based approaches, could enhance its therapeutic potential. However, challenges remain, including long-term safety, targeted delivery, and immune responses. Further research is needed to evaluate GATA2 stability, potential off-target effects, and its interaction with dentinogenesis pathways to pave the way for its integration into regenerative dentistry.

In summary, GATA2 is critical in regulating the odontogenic differentiation potential and dentin formation capacity of SCAPs. The regulation of GATA2 depends on the m^6^A site located in the 3′UTR of GATA2 mRNA. Mechanistically, METTL3 is the enzyme that catalyzes the m^6^A modification in this region, further modulating the stability of GATA2. This ultimately affects the odontogenic differentiation and dentin formation ability of SCAPs. Our research has unveiled a novel m^6^A epigenetic regulatory pattern in SCAPs for dentin formation and suggests a potential molecular target for dental tissue regeneration.

## 4. Materials and Methods

### 4.1. Ethics Statement

All animal research was conducted under the rules approved by the Beijing Stomatological Hospital, Capital Medical University. All research on human mesenchymal stem cells followed the ISSCR “Guidelines for the Conduct of Human Embryonic Stem Cell Research” (Ethical Committee Agreement, Beijing Stomatological Hospital Ethics Review No. CMUSH-IRB-KJ-PJ-2023-06).

### 4.2. Cell Culture and Odontogenic Differentiation Induction

SCAPs were isolated from third molars as previously described [41]. Cells were cultured in Dulbecco’s Modified Eagle Medium (DMEM; Gibco, Grand Island, NY, USA) supplemented with 10% fetal bovine serum (FBS; Gibco) and 1% penicillin-streptomycin (Gibco) at 37 °C in a 5% CO_2_ atmosphere. For odontogenic differentiation, SCAPs were seeded in six-well plates and treated with odontogenic differentiation medium containing DMEM supplemented with 10 mM β-glycerophosphate, 50 μg/mL ascorbic acid, and 10 nM dexamethasone (Sigma-Aldrich, St. Louis, MO, USA). Medium was refreshed every 3 days.

### 4.3. ALP Activity Assay

ALP activity was measured to assess odontogenic differentiation. SCAPs were cultured in six-well plates and induced with odontogenic medium for 3 days. Cells were lysed using RIPA buffer (Beyotime, Shanghai, China), and the supernatant was collected after centrifugation. ALP activity was determined using an ALP Assay Kit (Beyotime) following the manufacturer’s instructions. Absorbance was measured at 405 nm, and ALP activity was normalized to total protein content, determined using a BCA Protein Assay Kit (Thermo Fisher Scientific, Waltham, MA, USA). Data were expressed as mean ± SD from three independent experiments.

### 4.4. Alizarin Red S Staining

Mineralized nodules were visualized using alizarin red S staining. SCAPs were fixed with 4% paraformaldehyde and stained with 2% alizarin red S solution (pH 4.2) (Sigma-Aldrich) for 15 min at room temperature. Stained nodules were imaged, and quantification was performed by dissolving the dye in 10% cetylpyridinium chloride (Sigma-Aldrich) and measuring absorbance at 562 nm.

### 4.5. Lentiviral Vector Construction and Transduction

Lentiviral vectors were constructed for GATA2 overexpression and knockdown. GATA2 cDNA, GATA2 shRNA, and METTL3 shRNA sequences were cloned into the pLenti-CMV-GFP or pLenti-U6-GFP vectors (Addgene, Watertown, MA, USA). Lentiviruses were produced by co-transfecting HEK293T cells with the target plasmid and packaging plasmids using Lipofectamine 2000 (Invitrogen, Carlsbad, CA, USA). SCAPs were transduced with lentiviruses in the presence of 8 μg/mL polybrene (Sigma-Aldrich) and selected using 2 μg/mL puromycin for 3 days (LV3 shRNA (Consh), 5′-CAGUACUUUUGUGUAGUACAA-3′; METTL3 shRNA (METTL3sh), 5′-GCTGCACTTCAGACGAATTAT-3′; GATA2 shRNA (GATA2sh), 5′-GTGCAAATTGTCAGACGACAA-3′).

### 4.6. RNA Extraction and Quantitative Real-Time PCR (qPCR)

Total RNA was extracted using TRIzol reagent (Invitrogen) following the manufacturer’s instructions. Reverse transcription was performed using the PrimeScript RT reagent kit (Takara, Tokyo, Japan). qPCR was conducted using SYBR Green Master Mix (Applied Biosystems, Foster City, CA, USA) on an ABI 7500 system. GAPDH was used as an internal control, and relative expression levels were calculated using the 2^−ΔΔCt^ method. Primer sequences are listed in Supplementary Table S1.

### 4.7. Western Blot Analysis

Total protein was extracted from SCAPs using RIPA lysis buffer (Beyotime) supplemented with protease and phosphatase inhibitors (Roche, Basel, Switzerland). Protein concentrations were determined using a BCA Protein Assay Kit (Thermo Fisher Scientific). Proteins were separated by SDS-PAGE and transferred onto PVDF membranes (Merck Millipore, Billerica, MA, USA). Membranes were blocked with 5% non-fat milk and incubated overnight at 4 °C with primary antibodies against GATA2, DSPP, MEPE, and GAPDH (Abcam, Cambridge, MA, USA). After washing, membranes were incubated with HRP-conjugated secondary antibodies and visualized using an ECL reagent (Thermo Fisher Scientific).

### 4.8. RNA Methylation and m^6^A Site Mutations

To investigate m^6^A modification in the 3′UTR of GATA2, m^6^A site mutations were introduced. Wild-type GATA2 (WT-GATA2) and m^6^A site-mutated GATA2 (Mut-GATA2) sequences were cloned into the pCDH lentiviral vector (Clontech, Mountain View, CA, USA) to generate overexpression vectors. The accuracy of constructs was verified by Sanger sequencing. SCAPs were transduced with these constructs, and overexpression efficiency was confirmed by qPCR and Western blot.

### 4.9. RNA Stability Assay

RNA stability was evaluated using Actinomycin D assays. SCAPs transduced with WT-GATA2 or Mut-GATA2 were seeded at 80% confluence and treated with 2 μg/mL Actinomycin D (Sigma-Aldrich). RNA was extracted at 0, 3, and 6 h post-treatment using TRIzol reagent (Invitrogen). RNA expression levels were determined by qPCR, and stability was calculated using the 2^−ΔΔCt^ method.

### 4.10. Methylated RNA Immunoprecipitation Quantitative PCR (MeRIP-qPCR)

For MeRIP-qPCR, total RNA was fragmented and immunoprecipitated with m^6^A-specific antibodies (A19841, Abclonal, Wuhan, China). Enriched RNA was reverse transcribed using the PrimeScript RT reagent kit (Takara). Quantitative PCR was performed to measure m^6^A-enriched regions of GATA2 using primers specific to the 3′UTR. Primers are listed in Supplementary Table S1.

### 4.11. In Vivo Dentin Formation Assay

To evaluate dentin formation in vivo, adenoviral vectors (AAV9) carrying GATA2 or control sequences (produced by Genechem Co., Ltd., Shanghai, China) were injected into the buccal region of the first molar in 8-week-old mice. Mice were randomly divided into two groups (AAV9-GATA2 and AAV9-Control), with 8 mice per group. Adenoviral injections (1 μL per injection, 7 × 109 PFU) were administered once per week for three weeks over a four-week period. On day 28, teeth were harvested and fixed in 4% paraformaldehyde for histological analysis. Samples were embedded in paraffin, sectioned, and subjected to H&E, Masson’s trichrome, safranin O, and toluidine blue staining, which were performed according to the manufacturer’s instructions (Solarbio, Beijing, China).

### 4.12. Immunofluorescence Staining

Samples were fixed in 4% paraformaldehyde, deparaffinized, and subjected to antigen retrieval using citrate buffer (pH 6.0) at 95 °C for 15 min. Sections were blocked with 5% bovine serum albumin (BSA) for 1 h and incubated overnight at 4 °C with primary antibodies against DSPP (Bioss, Beijing, China). The next day, samples were washed and incubated with Alexa Fluor 488-conjugated secondary antibodies (Invitrogen, Waltham, MA, USA) for 1 h at room temperature. Nuclei were counterstained with DAPI (Beyotime, Nanjing, China) for 5 min. Fluorescence images were captured using a confocal microscope (Leica, Wetzlar, Germany).

### 4.13. Statistical Analysis

Statistical analyses were conducted using SPSS version 22 statistical software. In vitro experiments were independently repeated three times. In the case of in vivo experiments, six samples were utilized in each group for statistical analysis. A two-tailed Student’s *t*-test was employed for comparisons between two groups, while one-way ANOVA, followed by Bonferroni’s post hoc comparisons, was used for comparisons involving three or more groups. *p*-values below 0.05 were considered statistically significant.

## Data Availability

The datasets generated during the current study are available from the corresponding author upon reasonable request.

## Supplementary Materials

The following supporting information can be downloaded at: https://www.mdpi.com/article/10.3390/ijms26072920/s1.
