# Supplementary material for: m6A-Modified GATA2 Enhances Odontogenic Differentiation in Stem Cells from the Apical Papilla
Source: Int J Mol Sci. 2025 Mar 24;26(7):2920. doi: 10.3390/ijms26072920 (PMC11988903; doi:10.3390/ijms26072920)
Supplement: Supplementary file 1 [file ijms-26-02920-s001.zip › ijms-3506655-supplementary.pdf]

Supplementary information

GATA2-Mediated m6A Modification Enhances Odontogenic Differentiation in SCAPs

Haoqing Yang[1,2#], Fengning Yuan[1,3#], Zhipeng Fan* [2,3*]

1 Outpatient Department of Oral and Maxillofacial Surgery, School of Stomatology, Capital Medical University, Beijing, China
2 Laboratory of Molecular Signaling and Stem Cells Therapy, Beijing Key Laboratory of Tooth Regeneration and Function Reconstruction, Capital Medical University School of Stomatology, No. 4 Tiantanxili, Dongcheng District, Beijing, China
3 Beijing Laboratory of Oral Health, Capital Medical University, Beijing, China

[#] Haoqing Yang and Fengning Yuan contributed equally to this work.

*Correspondence:
Zhipeng Fan
zpfan@ccmu.edu.cn

Supplementary Table 1. Primers sequences used in the real-time RT-PCR

Gene Symbol	Primer Sequences (5'—3")
GAPDH-F	CGGACCAATACGACCAAATCCG
GAPDH-R	AGCCACATCGCTCAGACACC
GATA2-F	ACTGACGGAGAGCATGAAGAT
GATA2-R	CCGGCACATAGGAGGGGTA
Mettl3-F	TTGTCTCCAACCTTCCGTAGT
Mettl3-R	CCAGATCAGAGAGGTGGTGTAG
ALP-F	AAACTGGGGCCTGAGATACC
ALP-R	GCTTTCTTGGCCCGATTCAT
DMP1-F	CGTGGACAAAGAAGATAGCAACTCCACG
DMP1-R	TTCCGGCTCTCTATCTCAATGTTT
MEPE-F	GGCCAGTGACTGCGATTAAAC
MEPE-R	CCTTCGAGTGTGCTTTAGCAT
GATA2-m6A-F	TGGAAAAGAGCTGGGATCCC
GATA2-m6A-R	GGATCCAGACAGCCACAGTA

Vector

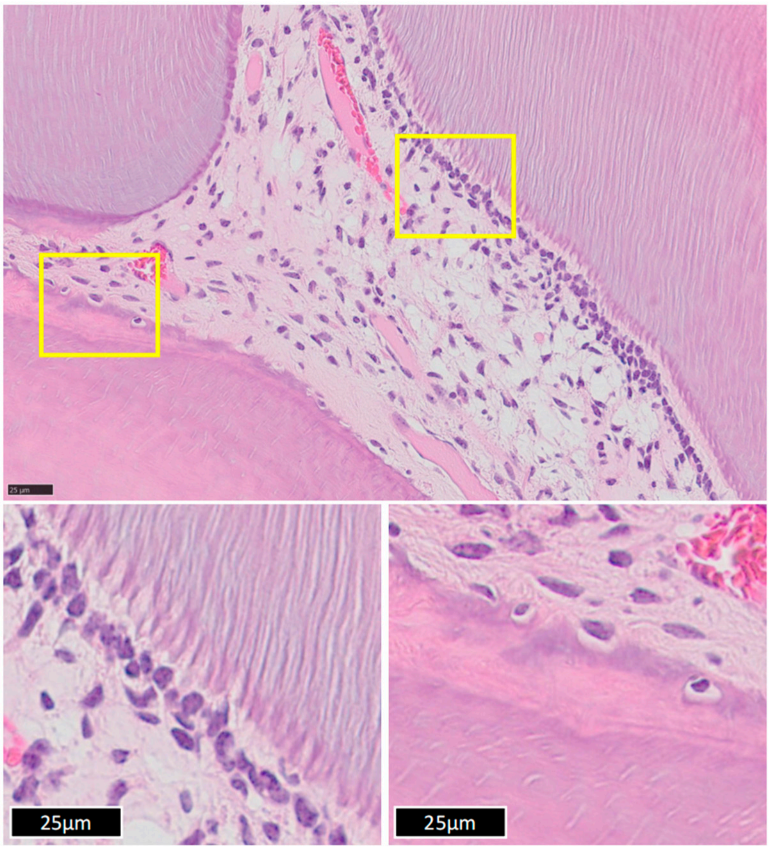

GATA2

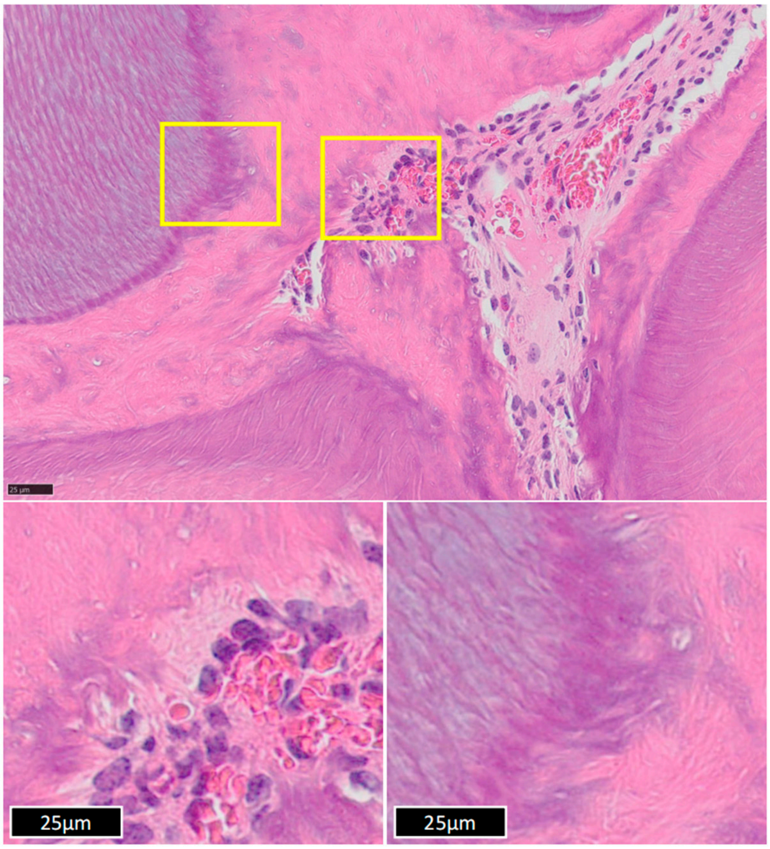

Supplementary Figure 1. Histological analysis of odontoblast and dentin matrix organization in the Vector and GATA2 groups.

Representative H&E-stained images showing the dentin-pulp interface in the Vector and GATA2 groups. In the Vector group, odontoblasts (highlighted in yellow boxes) are aligned in a single layer along the dentin surface, forming a well-organized structure. In contrast, the GATA2 group exhibits disrupted odontoblast alignment, with an increased number of odontoblast-like cells accumulating at the dentin-pulp interface. Additionally, dentin matrix deposition appears denser in the GATA2 group, potentially indicating increased tertiary dentin formation. Higher-magnification images (bottom panels) provide a detailed view of these cellular and matrix differences. Scale bars: 25 μm.

Supplementary Data 1. Identification of GATA2 Binding Site

>NM_001145661.2 Homo sapiens GATA binding protein 2 (GATA2), transcript variant 1, mRNAACTGGGTCAAGCACAGCCCTGAGCGGCCGCGTGTCCGAGGCCCAGGTGCCCTCTAGAG
CCCTGTAGTTCCTGCCCCTCTCTGCCCCTCTCGGCTCCTGCTGTTCCGCCGCTGTCGTCCGAACC
ATCCCAACCCCCAGTCCACCCAGACAGCGCCCGAGCTAGGGGAGGGAACGGTCTGGGAGTCG
GCAGCTGGCGCCAGGGCGGCCGGAGGATGCCGAGGGGCCGGAGCCGGGAGGGCCCGAGGC
CGAGGCGCACTCTACCCCCAGCTCCTACCCTGTAAGCCCCGCCAGCCTCCGGACGTGCTGTCC
CTGGGCCCGTCGCCCTCGGGGCTCCCGCCGGAACTCCTTCACTCTCAGAGGCCGAGTCCCTCC
CCTCCCCACGGCTGCGTGTGGCCGTTGCCGTCTGCACCCAGACCCTGAGCCGCCGCCGCCGGC
CATGGAGGTGGCGCCCGAGCAGCCGCGCTGGATGGCGCACCCGGCCGTGCTGAATGCGCAGC
ACCCCGACTCACACCACCCGGGCCTGGCGCACAACTACATGGAACCCGCGCAGCTGCTGCCTC
CAGACGAGGTGGACGTCTTCTTCAATCACCTCGACTCGCAGGGCAACCCCTACTATGCCAACCC
CGCTCACGCGCGGGCGCGCGTCTCCTACAGCCCCGCGCACGCCCGCCTGACCGGAGGCCAGA
TGTGCCGCCCACACTTGTTGCACAGCCCGGGTTTGCCCTGGCTGGACGGGGGCAAAGCAGCCC
TCTCTGCCGCTGCGGCCCACCACCACAACCCCTGGACCGTGAGCCCCTTCTCCAAGACGCCACT
GCACCCCTCAGCTGCTGGAGGCCCTGGAGGCCCACTCTCTGTGTACCCAGGGGCTGGGGGTGG
GAGCGGGGGAGGCAGCGGGAGCTCAGTGGCCTCCCTCACCCCTACAGCAGCCCACTCTGGCT
CCCACCTTTTCGGCTTCCCACCCACGCCACCCAAAGAAGTGTCTCCTGACCCTAGCACCACGGG
GGCTGCGTCTCCAGCCTCATCTTCCGCGGGGGGTAGTGCAGCCCGAGGAGAGGACAAGGACG
GCGTCAAGTACCAGGTGTCACTGACGGAGAGCATGAAGATGGAAAGTGGCAGTCCCCTGCGCC
CAGGCCTAGCTACTATGGGCACCCAGCCTGCTACACACCACCCCATCCCCACCTACCCCTCCTA
TGTGCCGGCGGCTGCCCACGACTACAGCAGCGGACTCTTCCACCCCGGAGGCTTCCTGGGGGG
ACCGGCCTCCAGCTTCACCCCTAAGCAGCGCAGCAAGGCTCGTTCCTGTTCAGAAGGCCGGGA
GTGTGTCAACTGTGGGGCCACAGCCACCCCTCTCTGGCGGCGGGACGGCACCGGCCACTACCT
GTGCAATGCCTGTGGCCTCTACCACAAGATGAATGGGCAGAACCGACCACTCATCAAGCCCAA
GCGAAGACTGTCGGCCGCCAGAAGAGCCGGCACCTGTTGTGCAAATTGTCAGACGACAACCAC
CACCTTATGGCGCCGAAACGCCAACGGGGACCCTGTCTGCAACGCCTGTGGCCTCTACTACAA
GCTGCACAATGTTAACAGGCCACTGACCATGAAGAAGGAAGGGATCCAGACTCGGAACCGGA
AGATGTCCAACAAGTCCAAGAAGAGCAAGAAAGGGGCGGAGTGCTTCGAGGAGCTGTCAAAG
TGCATGCAGGAGAAGTCATCCCCCTTCAGTGCAGCTGCCCTGGCTGGACACATGGCACCTGTG
GGCCACCTCCCGCCCTTCAGCCACTCCGGACACATCCTGCCCACTCCGACGCCCATCCACCCCT
CCTCCAGCCTCTCCTTCGGCCACCCCCACCCGTCCAGCATGGTGACCGCCATGGGCTAGGGAA
CAGATGGACGTCGAGGACCGGGCACTCCCGGGATGGGTGGACCAAACCCTTAGCAGCCCAGC
ATTTCCCGAAGGCCGACACCACTCCTGCCAGCCCGGCTCGGCCCAGCACCCCCTCTCCTGGAG
GGCGCCCAGCAGCCTGCCAGCAGTTACTGTGAATGTTCCCCACCGCTGAGAGGCTGCCTCCGC
ACCTGACCGCTGCCCAGGTGGGGTTTCCTGCATGGACAGTTGTTTGGAGAACAACAAGGACAA
CTTTATGTAGAGAAAAGGAGGGGACGGGACAGACGAAGGCAACCATTTTTAGAAGGAAAAAG
GATTAGGCAAAAATAATTTATTTTGCTCTTGTTTCTAACAAGGACTTGGAGACTTGGTGGTCTGA
GCTGTCCCAAGTCCTCCGGTTCTTCCTCGGGATTGGCGGGTCCACTTGCCAGGGCTCTGGGGGC
AGATTTGTGGGGACCTCAGCCTGCACCCTCTTCTCCTCTGGCTTCCCTCTCTGAAATAGCCGAAC
TCCAGGCTGGGCTGAGCCAAAGCCAGAGTGGCCACGGCCCAGGGAGGGTGAGCTGGTGCCTG

CTTTGACGGGCCAGGCCCTGGAGGGCAGAGACAATCACGGGCGGTCCTGCACAGATTCCCAG
GCCAGGGCTGGGTCACAGGAAGGAAACAACATTTTCTTGAAAGGGGAAACGTCTCCCAGATCG
CTCCCTTGGCTTTGAGGCCGAAGCTGCTGTGACTGTGTCCCCTTACTGAGCGCAAGCCACAGCC
TGTCTTGTCAGGTGGACCCTGTAAATACATCCTTTTTCTGCTAACCCTTCAACCCCCTCGCCTCCT
ACTCTGAGACAAAAGAAAAAATATTAAAAAAATGCATAGGCTTAACTCGCTGATGAGTTAATTG
TTTTATTTTTAAACTCTTTTTGGGTCCAGTTGATTGTACGTAGCCACAGGAGCCCTGCTATGAAAG
GAATAAAACCTACACACAAGGTTGGAGCTTTGCAATTCTTTTTGGAAAAGAGCTGGGATCCCAC
AGCCCTAGTATGAAAGCTGGGGGTGGGGAGGGGCCTTTGCTGCCCTTGGTTTCTGGGGGCTGG
TTGGCATTTGCTGGCCTGGCAGGGGGTGAAGGCAGGAGTTGGGGGCAGGTCAGGACCA**GGAC**
CCAGGGAGAGGCTGTGTCCCTGCTGGGGTCTCAGGTCCAGCTTTACTGTGGCTGTCTGGATCCT
TCCCAAGGTACAGCTGTATATAAACGTGTCCCGAGCTTAGATTCTGTATGCGGTGACGGCGGGG
TGTGGTGGCCTGTGAGGGGCCCCTGGCCCAGGAGGAGGATTGTGCTGATGTAGTGACCAAGTG
CAATATGGGCGGGCAGTCGCTGCAGGGAGCACCACGGCCAGAAGTAACTTATTTTGTACTAGT
GTCCGCATAAGAAAAAGAATCGGCAGTATTTTCTGTTTTTATGTTTTATTTGGCTTGTTTTATTTTG
GATTAGTGAACTAAGTTATTGTTAATTATGTACAACATTTATATATTGTCTGTAAAAAATGTATGC
TATCCTCTTATTCCTTTAAAGTGAGTACTGTTAAGAATAATAAAATACTTTTTGTGAA

GGAC： **binding site**
